# The role of AFAP1-AS1 in mitotic catastrophe and metastasis of triple-negative breast cancer cells by activating the PLK1 signaling pathway

**DOI:** 10.32604/or.2023.028256

**Published:** 2023-05-24

**Authors:** SHUIZHONG CEN, XIAOJIE PENG, JIANWEN DENG, HAIYUN JIN, ZHINAN DENG, XIAOHUA LIN, DI ZHU, MING JIN, YANWEN ZHU, PUSHENG ZHANG, YUNFENG LUO, HONGYAN HUANG

**Affiliations:** 1 Department of Spinal Surgery, Orthopedic Medical Center, Zhujiang Hospital, Southern Medical University, Guangzhou, 510280, China; 2 Department of Critical Care Medicine, Nanfang Hospital, Southern Medical University, Guangzhou, 510515, China; 3 Department of Breast Surgery, Zhujiang Hospital, Southern Medical University, Guangzhou, 510280, China; 4 Department of Gynecology and Obstetrics, Southern Hospital Taihe Branch, Southern Medical University, Guangzhou, 510540, China; 5 Department of Clinical Medicine, Nanshan Class, Guangzhou Medical University, Guangzhou, 511436, China; 6 Department of Gastroenterology, Shenzhen Hospital, Southern Medical University, Shenzhen, 518110, China

**Keywords:** TNBC, AFAP1-AS1, Mitotic catastrophe, Metastasis, PLK1

## Abstract

Triple-negative breast cancer (TNBC) is characterized by fast growth, high metastasis, high invasion, and a lack of therapeutic targets. Mitosis and metastasis of TNBC cells are two important biological behaviors in TNBC malignant progression. It is well known that the long noncoding RNA *AFAP1-AS1* plays a crucial role in various tumors, but whether *AFAP1-AS1* is involved in the mitosis of TNBC cells remains unknown. In this study, we investigated the functional mechanism of AFAP1-AS1 in targeting Polo-like Kinase 1 (PLK1) activation and participating in mitosis of TNBC cells. We detected the expression of *AFAP1-AS1* in the TNBC patient cohort and primary cells by *in situ* hybridization (ISH), northern blot, fluorescent *in situ* hybridization (FISH) and cell nucleus/cytoplasm RNA fraction isolation. High AFAP1-AS1 expression was negatively correlated with overall survival (OS), disease-free survival (DFS), metastasis-free survival (MFS) and recurrence-free survival (RFS) in TNBC patients. We explored the function of *AFAP1-AS1* by transwell, apoptosis, immunofluorescence (IF) and patient-derived xenograft (PDX) models *in vitro* and *in vivo*. We found that *AFAP1-AS1* promoted TNBC primary cell survival by inhibiting mitotic catastrophe and increased TNBC primary cell growth, migration and invasion. Mechanistically, *AFAP1-AS1* activated phosphorylation of the mitosis-associated kinase PLK1 protein. Elevated levels of *AFAP1-AS1* in TNBC primary cells increased PLK1 pathway downstream gene expression, such as CDC25C, CDK1, BUB1 and TTK. More importantly, *AFAP1-AS1* increased lung metastases in a mouse metastasis model. Taken together, *AFAP1-AS1* functions as an oncogene that activates the PLK1 signaling pathway. *AFAP1-AS1* could be used as a potential prognostic marker and therapeutic target for TNBC.

## Introduction

It is well known that breast cancer (BC) has become the highest cause of morbidity of women worldwide. Although various therapies have been utilized in breast cancer, such as surgery, chemotherapy, endocrine therapy, targeted therapy, and radiation therapy, the overall survival rate has not been improved [1,2]. TNBC is a highly heterogeneous tumor, and rapid growth and early metastasis are the main characteristics of TNBC development [3,4]. Due to the lack of expression of hormone (estrogen or progesterone) receptors and human epidermal growth factor receptor 2 (HER-2), TNBC lacks definite therapeutic targets. Therefore, intracellular biomolecules are good options to halt TNBC growth and progression. Further studies on TNBC progression are necessary to search for more effective therapeutic strategies to treat the disease [5].

Mitotic catastrophe is a very important and irreversible mechanism of TNBC cell death. Abnormal mitosis is highly heterogeneous and results in TNBC refractoriness in previous treatments [6,7].

Mitotic catastrophe is caused by abnormal mitosis and mitotic arrest and results in any of three irreversible cell fates: irreversible cell senescence, death during mitosis and irreversible death following mitosis [8].

Long noncoding RNAs (lncRNAs) are a class of nonprotein coding RNAs that are longer than 200 nucleotides in length and are closely connected with a variety of human diseases, particularly cancer [9]. lncRNAs can regulate the biological behaviors of TNBC as oncogenes or anti-oncogenes to activate or inhibit cancer-associated signaling pathways through various functional mechanisms [10]. Many studies have suggested that lncRNAs participate in TNBC survival [11], glycolysis [12], metastasis [13], death [14] and drug resistance [15]. However, the molecular mechanism by which lncRNAs influence TNBC cell death is still unknown. *AFAP1-AS1* has been reported to be aberrantly upregulated in many malignant tumors, such as lung cancer [16], esophageal adenocarcinoma [17], and TNBC [18]. Previous work suggested that high expression of *AFAP1-AS1* was associated with the outcome of TNBC patients and inhibited TNBC cell death [19]. However, the relationship between *AFAP1-AS1* and mitosis in TNBC is unknown.

In this study, parallel to previous studies, we found that *AFAP1-AS1* was upregulated in TNBC tissue samples and primary cells compared to peri-tumor tissues and benign primary cells, and *AFAP1-AS1* upregulation was closely correlated with poor overall and disease-free survival of TNBC patients. *AFAP1-AS1* knockout induced mitotic catastrophe and apoptosis in TNBC primary cells *in vitro*. Mechanistically, silencing *AFAP1-AS1* inhibited PLK1 phosphorylation and thereby promoted mitotic catastrophe in TNBC primary cells and tumor metastasis *in vitro*. Together, our results show that *AFAP1-AS1* is a lncRNA involved in the mitosis process of TNBC and indicate the significance of further investigation of AFAP1-AS1 in targeted therapy of TNBC.

## Materials and Methods

### Patients and tissue samples

A total of 368 TNBC patients’ clinical tissue samples were fixed in formalin and collected from the Department of Breast Surgery, Zhujiang Hospital, Southern Medical University (Guangzhou, China) from 2012 to 2022. The overall survival of these 368 TNBC samples was followed up with a median period of 78 months. We randomly selected 100 TNBC patients from the 368 TNBC patients, and their tissue RNA was extracted and preserved at −80°C from 2017 to 2022. Five cases of fresh TNBC tumors and three cases of fibroadenoma (FA) tumors were immediately collected post-operation in Zhujiang Hospital from 2020-2022, and detailed information is listed in Suppl. Table S1. All tissue samples enrolled in this study signed informed written consent documents. All protocols were approved by the Research Scientific Ethics Committee of the Zhujiang Hospital, Southern Medical University (Guangzhou, China).

### Northern blot

This assay was used to detect the expression of *AFAP1-AS1* in different breast tumor primary cells or the efficiency of *AFAP1-AS1* knockdown. Total RNA that was extracted from breast tumor primary cells was subjected to Northern blot assays as per the protocol provided by Roche (Basel, Switzerland), where we used digoxin-labeled *AFAP1-AS1* probes (50–100 μM) to hybridize overnight at room temperature. Probe detection was performed using a digoxin Luminescent Detection Kit for Nucleic Acids (Roche, Basle, Switzerland).

### ISH and FISH

A total of 368 TNBC tissues and paired peri-tumors were fixed and sectioned, and the sections were dewaxed and rehydrated. Then, the tissues were hybridized in prehybridization solution at 65°C overnight. *AFAP1-AS1*-labeled 5′digoxin was purchased from Exiqon (Beijing, China). After hybridization, the tissues were incubated with anti-digoxin antibody from Abcam (Cambridge, England) at 4°C overnight. Finally, the nitro blue tetrazolium (NBT)/5-bromo-4-chlor-o-3-indolyl phosphate (BCIP) substrate was used for *AFAP1-AS1* staining, and nuclear fast red was used for nuclear staining. The *AFAP1-AS1* staining results were calculated on both the intensity and proportion of positive cells by two pathologists [20]. A staining index (SI) >6 was distributed to the *AFAP1-AS1* high expression group, and ≤6 was distributed to the *AFAP1-AS1* low expression group. On the basis of the SI score distribution for the *AFAP1-AS1* expression level, we analyzed the correlation between *AFAP1-AS1* expression and overall survival, disease-free survival, recurrence-free survival, and metastasis-free survival.

For FISH, we used a FISH kit (Ribo^TM^, Guangzhou, China) according to the manufacturer’s instructions. In the kit, an 18S probe was used as a cytoplasmic control, and U6 was used as a nuclear control.

### Primary cell extraction and culture

Breast cancer cells from TNBC tissue samples were obtained from surgery, and benign breast cells from FA tissue samples were obtained from surgery. The tissues were cut up and digested by collagenase type III at 37°C with vibration for 2–3 h in DMEM with 10% FBS and then filtered through a 70 μm strainer. The tumor cells were collected by centrifugation at 1800 rpm for 8 min. The primary tumor cells were cultured in DMEM with 20% FBS. After three days, the nonadherent cells were removed by fresh medium, and second- or third-passage primary tumor cells were used for individual experiments. More importantly, the purity of primary tumor cells was validated by flow cytometry analysis, which showed that the primary tumor cells were positive for CD326 (EpCAM) and negative for FAP (>95%) [2].

### Flow cytometry analysis

This assay was used to analyze the expression of cell surface molecular markers. Cells were resuspended in PBS and stained with fluorescent-conjugated antibodies against CD326 and FAP for 30 min at room temperature. Specimens were analyzed by a BD Accuri C6 Flow cytometer, and at least three independent experiments were conducted in each analysis.

### Cell count kit-8 (CCK-8) assay

The Cell Count Kit-8 (MCE, American) was used to detect the the viability of TNBC primary cells. Brifely, 2000 cells treated with siRNAs per well were cultured into 96-well plates (Corning, NY, USA). Removing culture-medium after 48 h, and 10 μl CCK-8 reagent was added into each well at the time of harvest. The cells were incubated at 37°C for 1 h. Finally, at the time points, the absorbance at 450 nm was measured to detect the cell viability using the microplate reader (BioTek, USA). The results are representative of three independent experiments in triplicate.

### Apoptosis analysis

The indicated cells were pretreated with si-*AFAP1-AS1* (RiboBio, Guangzhou, China) for 48 h, subsequently trypsinized with 0.25% trypsin-EDTA and harvested by centrifugation in PBS. Apoptosis was detected using an Annexin V Apoptosis Detection Kit (Sigma, Germany). Briefly, cells were incubated with 100 μL binding buffer containing 5 μL of FITC-conjugated Annexin V antibody for 15 min at room temperature. After incubation, the cells were washed and resuspended in 200 μL of binding buffer containing 5 μL of propidium iodide staining solution and immediately analyzed by flow cytometry.

### Cell nucleus/cytoplasm RNA fraction isolation

We used the NE-PER^TM^ Nuclear and Cytoplasmic Extraction Kit (Thermo, American) to detect the ratio of *AFAP1-AS1* in the nucleus and cytoplasm. The expression of *AFAP1-AS1* in the nucleus and cytoplasm was measured by qRT‒PCR. U6 was used as the nuclear positive control, and 18S was used as the cytoplasm positive control.

### Cell migration and invasion assays

In this study, to explore the migration and metastasis abilities of TNBC primary cells, to imitate the cell barrier, we used Transwell chambers. In the invasion assay, a layer of Matrigel (BD Bioscience) was placed above the chamber. The indicated cells were pretreated with silnc-*AFAP1-AS1* (RiboBio, Guangzhou, China) for 48 h and subsequently trypsinized and washed in PBS. Two thousand cells were seeded into the upper chambers in DMEM with 3% FBS, and DMEM with 10% FBS was added to the lower chambers. After approximately 12 h, the chambers were collected and quantified by photographing 3 random fields.

### IF

Immunofluorescence staining of α-tubulin and γ-tubulin was performed to evaluate the effect of *AFAP1-AS1* knockdown on mitotic catastrophe in TNBC primary cells. The TNBC primary cells (case 5) were preprocessed by using si-NC and si-*AFAP1-AS1* transfection according to the protocol of Ribo^TM^, Guangzhou, China. Treated cells were treated as follows: cells were fixed in tissue fixative solution for 15 min, permeabilized for 10 min with 0.1% Triton X-100, blocked for 30 min with 1% BSA at RT, and then incubated with primary antibodies against α-tubulin (05-829X-555) and γ-tubulin (T3320) for 2 h at RT. The secondary antibodies were incubated for 1 h at RT. The cell nuclei were stained using DAPI solution. Finally, images were acquired by laser confocal microscopy (Leica Microsystems). We randomly selected 5 fields (approximately 300 cells) and statistically analyzed mitotic catastrophe in each group.

### PDX model in vivo

To explore the influence of *AFAP1-AS1* on TNBC tumorigenesis, we established one case PDX as follows; TNBC clinical specimens were obtained from TNBC patients who had tumors excised at Zhujiang Hospital, South Medical University (Guangzhou, China) in 2022. Detailed information is listed in Suppl. Table S1. The tumor was cut into small incisions and placed into the fourth pair of mammary fat pads of anesthetized four-week-old NOSCID. When the maximum diameter of PDX reached 5 mm at approximately 21 days, the two locked nucleic acids (LNAs) against *AFAP1-AS1* were intravenously injected with NOSCID once every week for approximately 6 weeks.

### Metastasis model in vivo

To establish a metastasis model, we transduced luciferase lentivirus into the TNBC cell line MDA-MB-231 and knocked out *AFAP1-AS1*. A total of 1 × 10^6^ cells were mixed in 200 μl of PBS and intravenously injected into the tail vein of Balb/c nude mice. After two months, the mice were anesthetized and imaged by the IVIS Lumina Imaging System, and the lung metastasis nodes were cut into sections and stained with HE.

### Statistical analysis

All statistical analyses were conducted by Student’s *t* test by using GraphPad Prism 5.0, and the results *in vitro* and *in vivo* were repeated in three independent experiments. The statistical analyses of the results *in vitro* and *in vivo* are separately presented as the mean ± SD and mean ± SEM.

## Results

### Aberrant upregulation of AFAP1-AS1 is associated with TNBC patient progression

**Figure 1:**
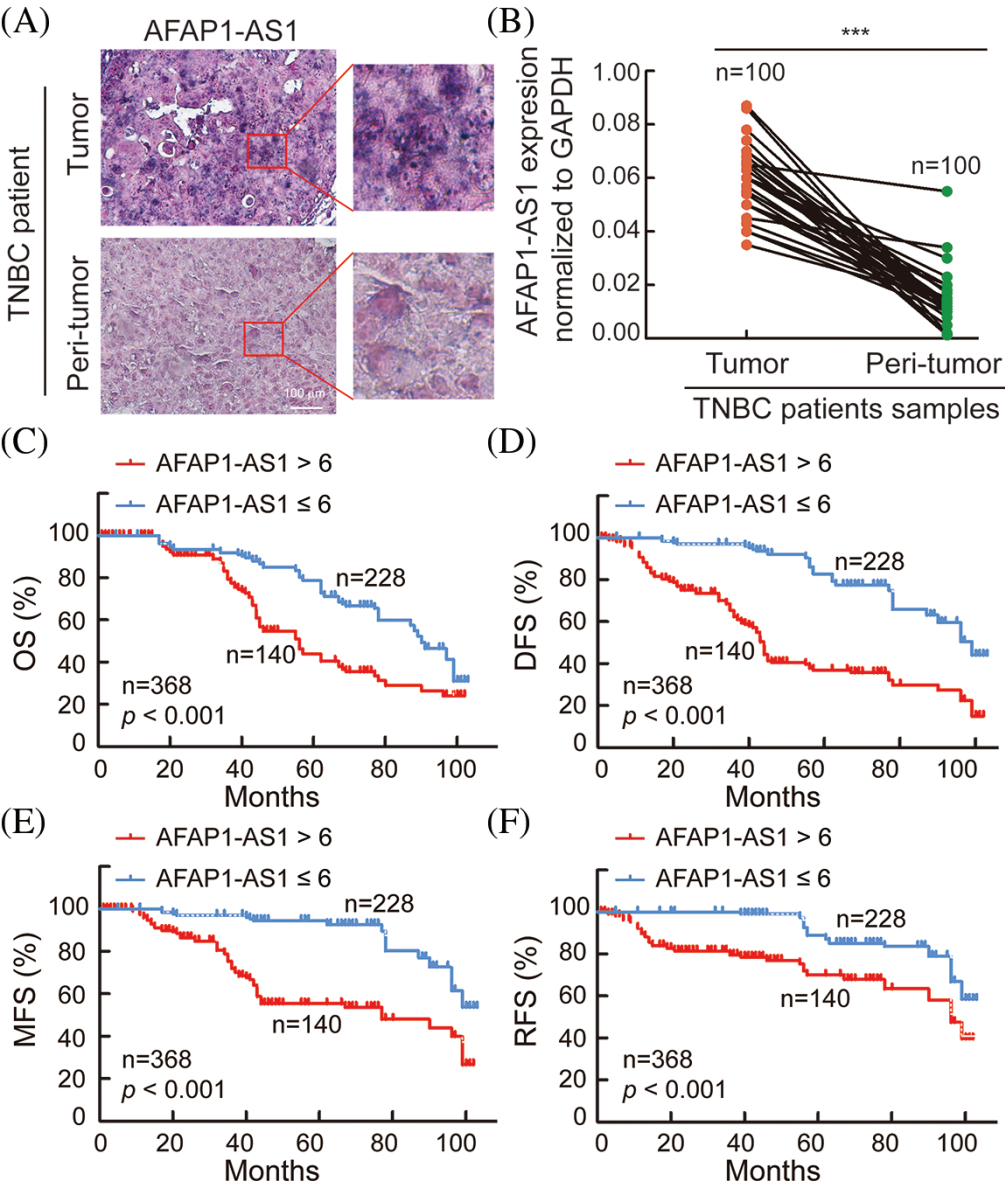
AFAP1-AS1 is highly expressed and associated with TNBC patient prognosis. (A) Representative ISH images showed that AFAP1-AS1 was upregulated in TNBC cancer tissues compared with peritumour tissues (scale bar = 100 μm). (B) In 100 TNBC tissue RNA samples, AFAP1-AS1 was also upregulated in cancer tissues compared with peritumour tissues by qRT‒PCR (***p < 0.001). (C) Based on the AFAP1-AS1 ISH results of 1A in 368 TNBC tumor sample sections, the patients with high AFAP1-AS1 expression (ISH scores > 6, *n* = 228) had poorer OS than those with low AFAP1-AS1 expression (ISH scores ≤ 6, *n* = 140) according to Kaplan‒Meier curves (***p < 0.001 by paired Student’s *t* tests, mean ± SEM). (D) Kaplan‒Meier curves showed that the patients with high AFAP1-AS1 expression had poorer DFS than those with low AFAP1-AS1 expression (***p < 0.001 by paired Student’s *t* tests, mean ± SEM). (E) Kaplan‒Meier curves showed that the patients with high AFAP1-AS1 expression had poorer MFS than those with low AFAP1-AS1 expression (***p < 0.001 by paired Student’s *t* tests, mean ± SEM). (F) Kaplan‒Meier curves showed that the patients with high AFAP1-AS1 expression had poorer RFS than those with low AFAP1-AS1 expression (***p < 0.001 by paired Student’s *t* tests, mean ± SEM).

In 368 pairs of TNBC patient tissue sections from our own cohort, we detected *AFAP1-AS1* expression in tumors and peritumor tissues via ISH. *AFAP1-AS1* expression was significantly higher in tumor tissues than in peritumor tissues (Fig. 1A). Meanwhile, we detected the *AFAP1-AS1* RNA level in 100 pairs of TNBC patient tissue RNA samples that were randomly selected from the 368 pairs of TNBC patient tissues by qRT‒PCR, and we obtained the same conclusion that *AFAP1-AS1* expression was increased in tumor compared to adjacent tumor tissues (Fig. 1B). In our 368 pairs of TNBC cohorts, high expression of *AFAP1-AS1* (ISH scores > 6) was closely related to poor prognosis in TNBC patients, and patients with higher *AFAP1-AS1* expression had poorer OS (Fig. 1C), DFS (Fig. 1D), MFS (Fig. 1E) and RFS (Fig. 1F). We analyzed the relationship between *AFAP1-AS1* and the clinicopathological features of 368 TNBC tissue samples and found that higher *AFAP1-AS1* expression was significantly related to tumor size, metastasis and Ki-67 level but was not associated with age, menopausal status, histological grade or recurrence (Table 1). Univariate and multivariate Cox proportional hazard analyses showed that *AFAP1-AS1* (*p* < 0.001) was a poor independent prognostic factor for OS and DFS (Suppl. Table S2). The above results showed that *AFAP1-AS1* may be a prognostic factor in TNBC patients’ malignant progression.

**Table 1:** Correlation of AFAP1-AS1 expression with the clinicopathologic status of 368 TNBC patients

Characteristics	*n*	AFAP1 expression		*p*
		>6	≤6	
Total	368	228	140	
Age (years)				0.655
≤40	132	84	48	
>40	236	144	92	
Menopausal status				0.591
Premenopausal	184	117	67	
Postmenopausal	184	111	73	
Tumor size (cm)				0.013
≤3	189	129	60	
>3	179	99	80	
Histological grade				0.133
Grade I/II	190	125	65	
Grade III	178	103	75	
Recurrence				0.424
Yes	75	50	25	
No	293	178	115	
Metastasis				0.048
Yes	93	66	27	
No	275	162	113	
Ki-67 level				0.038
≤15%	148	82	66	
>15%	220	146	74	

### In TNBC primary cells, AFAP1-AS1 is highly expressed and mainly located in the cytoplasm

**Figure 2:**
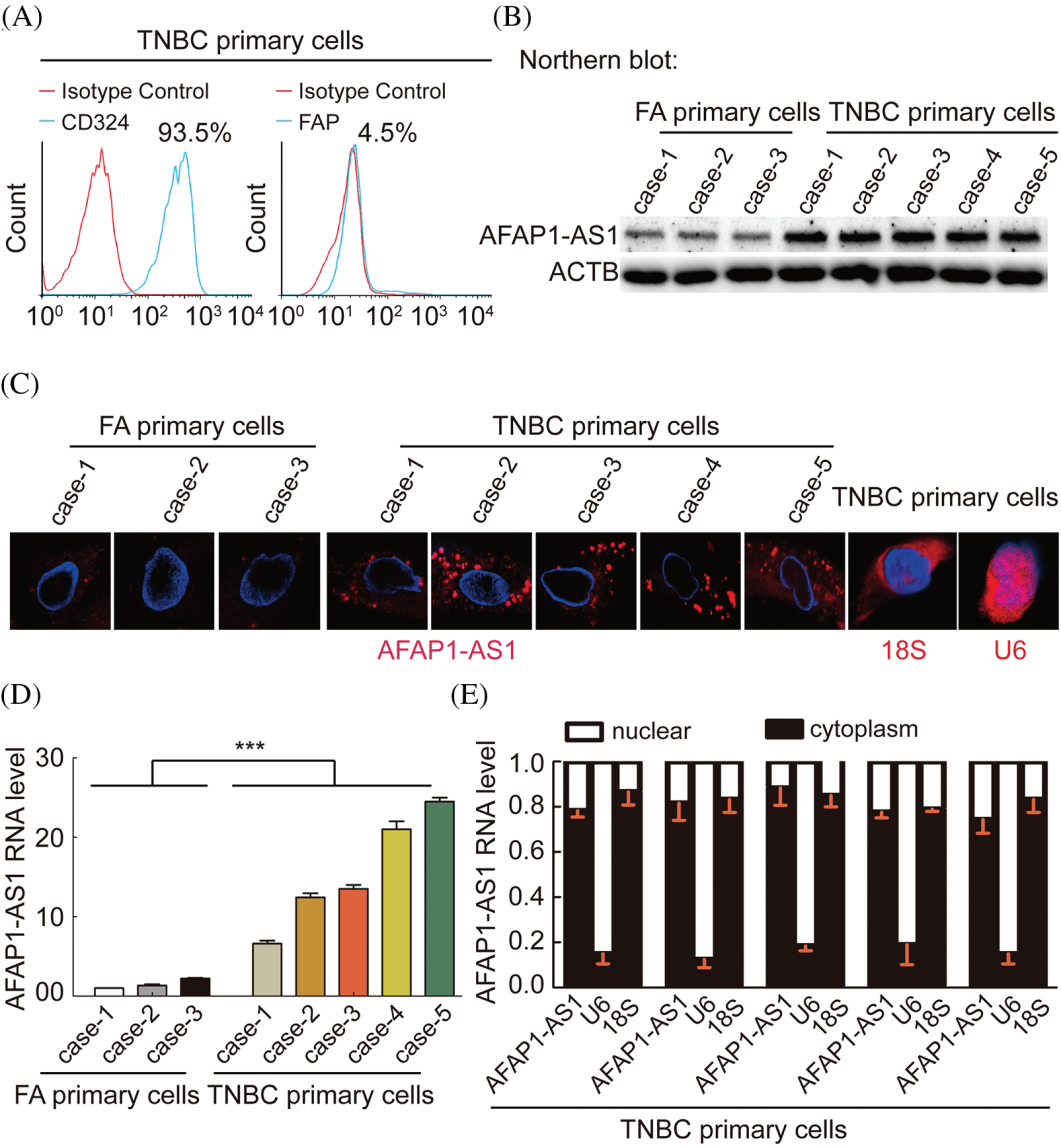
In TNBC primary cells, AFAP1-AS1 is highly expressed and mainly located in the cytoplasm. (A) The ratio of CD326 (epithelial marker) positive in extracted TNBC primary cells is up to 90%, and the ratio of FAP (stroma marker) positive in extracted TNBC primary cells is down to 5% by flow cytometry. (B) NB assays showed that AFAP1-AS1 is upregulated in 5 TNBC primary cell samples compared with 3 FA primary cell samples. (C) FISH assays showed that AFAP1-AS1 is upregulated in 5 TNBC primary cells compared with 3 FA primary cells and is mainly located in the cytoplasm. 18S and U6 were used as cytoplasmic and nuclear controls, respectively. (D) qRT‒PCR showed that AFAP1-AS1 was upregulated in 5 TNBC primary cell samples compared with 3 FA primary cell samples (***p < 0.001 by paired Student’s *t* tests, mean ± SEM). (E) Cell nuclear and cytoplasmic RNA fraction isolation assays showed that AFAP1-AS1 was mainly located in the cytoplasm, and 18S and U6 were used as the cytoplasmic and nuclear controls, respectively.

To explore the characterization, including the expression and cellular distribution of *AFAP1-AS1*, we successfully extracted 5 TNBC primary cell samples and 3 breast FA samples as a control group. The summary extraction procedure of primary cells was performed according to previous work [2], and the purity of primary tumor cells (epithelial cells) was over 90% by flow cytometry (Fig. 2A). Patient information for primary cells is shown in Suppl. Table S1.

We detected the *AFAP1-AS1* RNA levels in the above 8 breast tumor primary cells by Northern blot, FISH and qRT‒PCR. We also found that the expression of *AFAP1-AS1* was increased in TNBC cells compared to FA primary cells (Figs. 2B–2D). In FISH and qRT‒PCR following cytoplasm/nuclear fractions assays, the results showed that AFAP1-AS1 is mainly distributed in the cytoplasm of both TNBC and FA primary cells (Figs. 2C and 2E), using 18S and U6 as cytoplasm and nuclear positive control. These results suggest that AFAP1-AS1 of cytoplasm is higher in TNBC than breast benign primary cells.

### AFAP1-AS1 knockdown inhibited migration and invasion and induced mitotic catastrophe and apoptosis in TNBC primary cells in vitro

**Figure 3:**
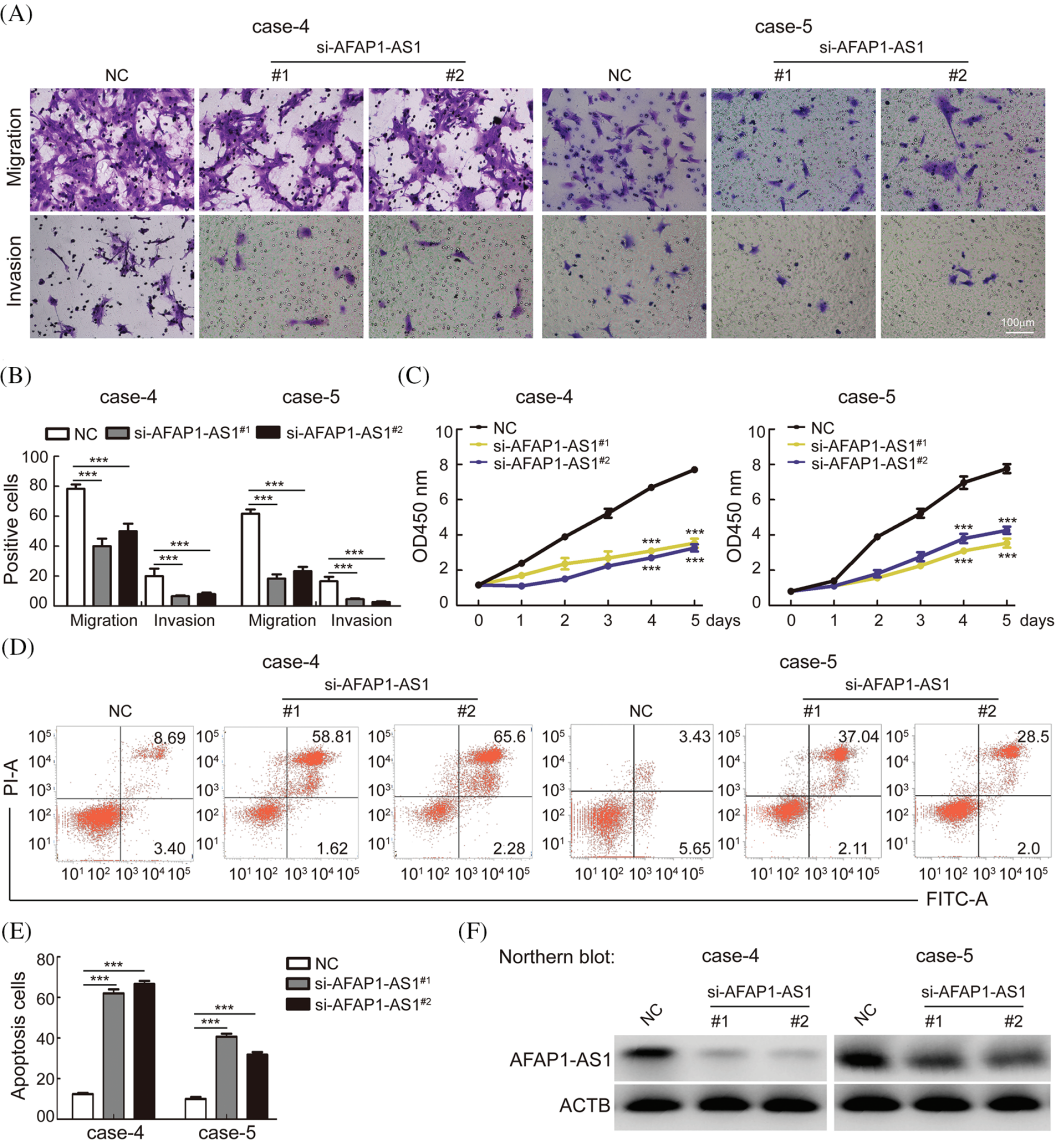
Knockdown of AFAP1-AS1 inhibited TNBC primary cell migration, invasion, and proliferation and induced apoptosis. (A) Representative images of AFAP1-AS1 knockdown-inhibited migration and invasion of TNBC primary cells (scale bar = 100 μm). (B) The statistical results of 3 A (***p < 0.001 by paired Student’s *t* tests, mean ± SD). (C) AFAP1-AS1 knockdown inhibited the proliferation of TNBC primary cells, as determined by CCK8 assay (***p < 0.001 by paired Student’s *t* tests, mean ± SD). (D) Representative images of AFAP1-AS1 knockdown-induced TNBC primary cell apoptosis by flow cytometry assay. (E) The statistical results of 3 D (***p < 0.001 by paired Student’s *t* tests, mean ± SD). (F) Efficiency of AFAP1-AS1 knockdown in TNBC primary cells by NB assays.

To explore the functional role of *AFAP1-AS1* in TNBC primary cells, we selected cases 4 and 5, in which the expression of *AFAP1-AS1* was highest among TNBC primary cells, as shown in Fig. 2. To study the possible role of *AFAP1-AS1* in case 4 and case 5 cells, two siRNAs against *AFAP1-AS1* were used. Then, a transwell assay showed that *AFAP1-AS1* knockdown remarkably inhibited the migration and invasion abilities of case 4 and case 5 cells (Figs. 3A and 3B). Regarding the proliferation ability of cells, the CCK8 assay indicated that *AFAP1-AS1* knockdown significantly inhibited the proliferation ability of case 4 and case 5 primary cells (Fig. 3C). Apoptosis assays showed that *AFAP1-AS1* knockdown obviously induced the death of case 4 and case 5 cells (Figs. 3D and 3E), suggesting that *AFAP1-AS1* was essential for the survival of TNBC cells. We detected the knockdown efficiency of *AFAP1-AS1* by Northern blot and qRT‒PCR assays, and the knockdown efficiency of *AFAP1-AS1* was up to 90% (Fig. 3F).

**Figure 4:**
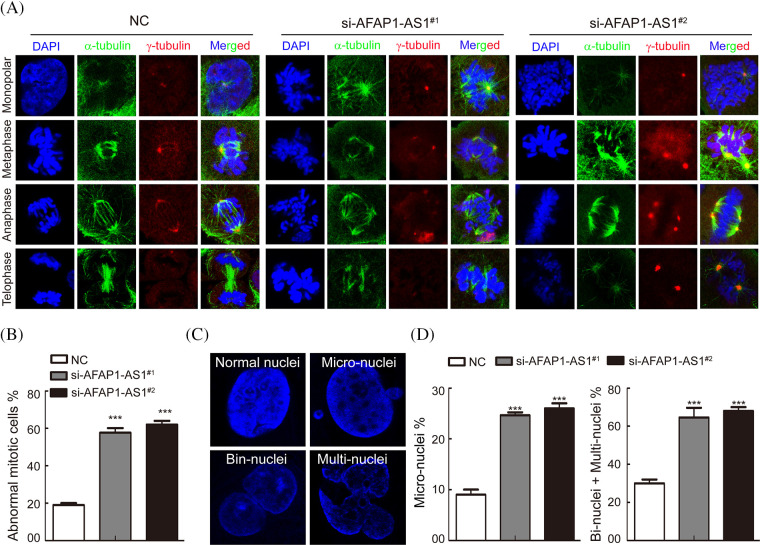
Knockdown of AFAP1-AS1 induced mitotic catastrophe in TNBC primary cells. (A) IF staining with α-tubulin and γ-tubulin in TNBC primary cells showed that AFAP1-AS1 knockdown induced mitotic catastrophe in the mitosis cycle. (B) The statistics of mitotic catastrophe cells in A (***p < 0.001 by paired Student’s *t* tests, mean ± SD). (C) Representative images of abnormal nuclei (micronuclei, bin-nuclei and multinuclei) stained with DAPI. (D) The statistics of abnormal nuclei (including micronuclei, bin nuclei and multinuclei) in C (***p < 0.001 by paired Student’s *t* tests, mean ± SD).

It is well known that a regular mitotic cycle is a key step in sustaining tumor cell survival [21]. To investigate the cellular events that triggered apoptosis upon *AFAP1-AS1* silencing, we detected mitosis in *AFAP1-AS1*-knockdown cells before they underwent cell death. Immunostaining of α-tubulin and γ-tubulin in case 5 primary cells by IF staining showed that *AFAP1-AS1* knockdown induced mitotic catastrophe occurrence in different mitosis phases (Fig. 4A). The statistical data showed that *AFAP1-AS1* knockdown led to approximately 60% mitotic catastrophe (Fig. 4B).

During mitotic catastrophe in cells, abnormal cell nuclei, including micronuclei, bin-nuclei, and multinuclei, eventually lead to mitotic catastrophe [22]. Then, we found that *AFAP1-AS1* knockdown increased the proportion of the above abnormal cell nuclei (Figs. 4C and 4D). These data reveal that *AFAP1-AS1* knockdown induces seriously abnormal mitosis and stimulates mitotic catastrophe in TNBC primary cells, leading to TNBC cell death.

### In TNBC primary cells, AFAP1-AS1 knockdown negatively regulated PLK1 phosphorylation

**Figure 5:**
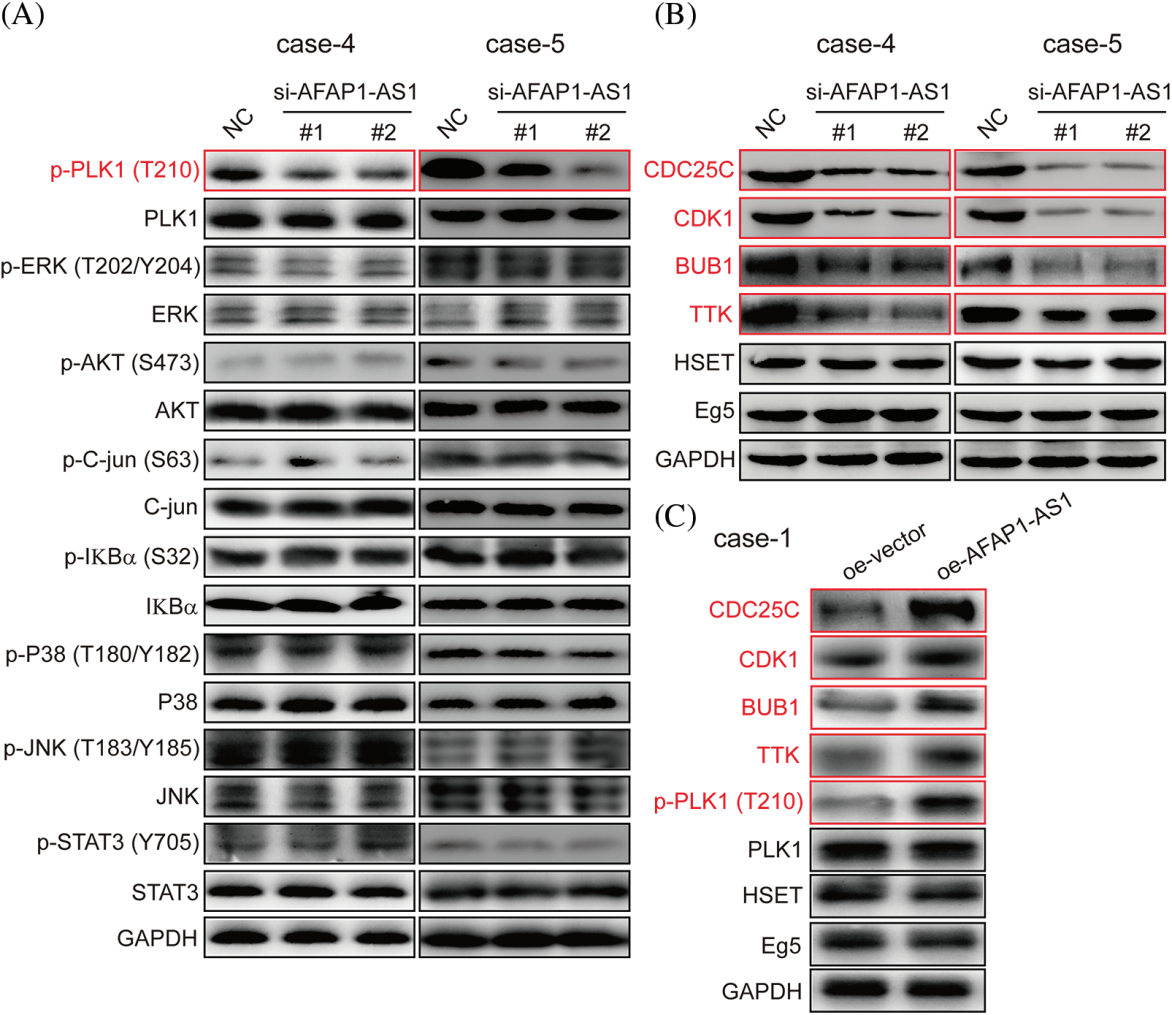
Knockdown of AFAP1-AS1 inhibited PLK1 phosphorylation and downstream gene expression. (A) AFAP1-AS1 knockdown significantly inhibited PLK1 phosphorylation rather than the phosphorylation of ERK, AKT, C-jun, IKBα, P38, JNK, and STAT3 in 2 TNBC primary cell lines by WB assay. (B) AFAP1-AS1 knockdown significantly inhibited the expression of PLK1 pathway downstream genes, including CCDC25C, CDK1, BUB1 and TTK. AFAP1-AS1 knockdown did not affect other mitosis-associated proteins, such as HSET and Eg5, in TNBC primary cells (case 4 and case 5) by WB assay. (C) AFAP1-AS1 was overexpressed in TNBC primary cells (case 1: low expression AFAP1-AS1), the expression of PLK1 pathway downstream genes, including CCDC25C, CDK1, BUB1 and TTK, was increased, and the expression of other mitosis-associated proteins, such as HSET and Eg5, was not changed.

In previous studies, the activation of the PLK1, MAPK/ERK, JNK/STAT, AKT, and NF-κB pathways was associated with the malignant biology of cancer cells [23]. The PLK1 signaling pathway has been reported to play crucial roles in mitotic catastrophe progression in most cancer cells [24]. To identify which of the above pathways participated in *AFAP1-AS1* function in TNBC primary cells, after knockdown of *AFAP1-AS1*, we examined the above possible signaling pathways in TNBC primary cells (case 4 and case 5) by Western-blot (WB). Our results showed that *AFAP1-AS1* knockdown decreased the expression of PLK1 phosphorylation in TNBC primary cells (case 4 and case 5). In addition, we found that the MAPK/ERK, JNK/STAT, AKT, and NF-κB pathways were not obviously changed (Fig. 5A). To further confirm that *AFAP1-AS1* activates PLK1 signaling, after *AFAP1-AS1* silencing or overexpression, we detected PLK1 downstream molecules, such as CDC25C, CDK1, BUB1, and TTK. The results showed that *AFAP1-AS1* knockdown decreased the expression of PLK1 downstream genes in TNBC primary cells (Fig. 5B). After *AFAP1-AS1* overexpression in FA primary cells (case 1), *AFAP1-AS1* overexpression increased the expression of PLK1 downstream genes (Fig. 5C). On the other hand, HSET, a mitosis-associated protein, has been linked to the mitosis of cancer cells [25]. Therefore, we examined whether *AFAP1-AS1* affects HSET and Eg5 expression and found that *AFAP1-AS1* did not influence HSET and Eg5 expression after *AFAP1-AS1* silencing or overexpression (Figs. 5B and 5C). These results suggested that *AFAP1-AS1* induced mitosis in TNBC primary cells by activating PLK1 signaling.

### AFAP1-AS1 mediates mitosis of TNBC primary cells through activation of the PLK1 pathway

**Figure 6:**
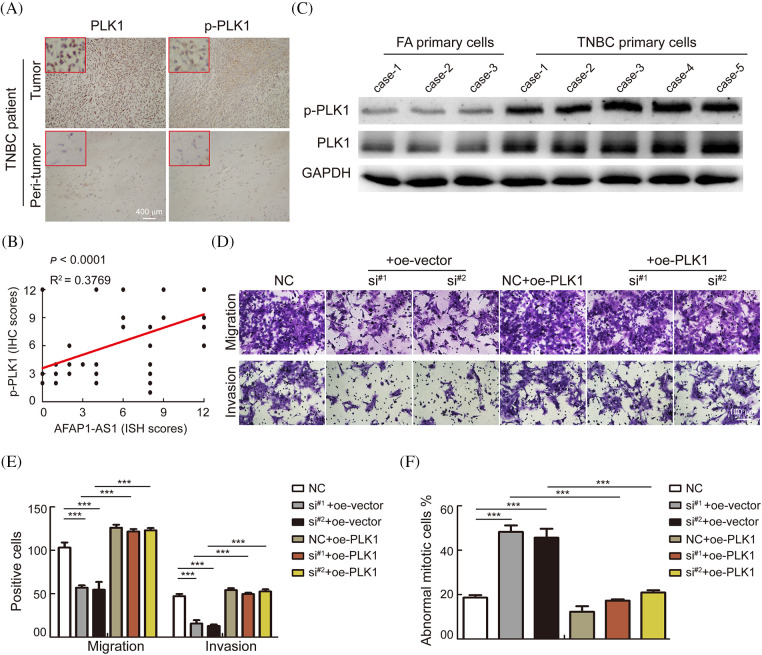
AFAP1-AS1 mediates the malignant biological function of TNBC primary cells through activation of the PLK1 pathway. (A) Representative images of IHC in serial tissue sections showed that PLK1 and p-PLK1 were upregulated in TNBC cancer tissues compared with peritumour tissues (scale bar = 400 μm). (B) The expression of AFAP1-AS1 was positively correlated with p-PLK1 in 368 TNBC tissues, R^2^ = 0.3769, *p* < 0.0001. (C) WB assays showed that PLK1 and p-PLK1 were upregulated in 5 TNBC primary cell samples compared with 3 FA primary cell samples. (D) Transwell assays showed that AFAP1-AS1 knockdown significantly inhibited migration and invasion abilities, and PLK1 overexpression rescued the inhibition of migration and invasion abilities by AFAP1-AS1 knockdown in TNBC primary cells (scale bar = 100 μm). (E) The statistics of positive cells about D (***p < 0.001 by paired Student’s *t* tests, mean ± SD). (F) The statistical analyses showed that AFAP1-AS1 knockdown significantly increased the abnormal mitotic cells, and PLK1 overexpression inhibited the augmentation of abnormal mitotic cells by AFAP1-AS1 knockdown in TNBC primary cells (***p < 0.001 by paired Student’s *t* tests, mean ± SD).

To further verify whether *AFAP1-AS1* promoted malignant progression of TNBC by activating the PLK1 pathway. We first detected the expression of PLK1 and p-PLK1 in 368 TNBC tissue sections and found that the expression in tumors was significantly higher than that in peritumor tissues by immunohistochemistry (IHC) (Fig. 6A). In the 368 TNBC patient tissue samples, the expression of *AFAP1-AS1* was positively correlated with p-PLK1 expression (Fig. 6B). In TNBC primary cells, we found that PLK1 and p-PLK1 were upregulated in TNBC primary cells compared with FA primary cells by WB assay (Fig. 6C). The above results indicated that the PLK1 pathway was activated in TNCB and positively correlated with *AFAP1-AS1* expression. In TNBC primary cell functional experiments, we found that *AFAP1-AS1* knockdown inhibited the migration and invasion abilities of TNBC primary cells, and *AFAP1-AS1* knockdown and PLK1 overexpression rescued migration and invasion abilities compared with *AFAP1-AS1* knockdown (Figs. 6D and 6E). At the same time, *AFAP1-AS1* knockdown and PLK1 overexpression decreased the number of abnormal mitotic cells compared with *AFAP1-AS1* knockdown (Fig. 6F). We concluded that the aberrant expression of *AFAP1-AS1* is an oncogenic gene that stimulates the PLK1 pathway.

### Knockdown of AFAP1-AS1 inhibited TNBC growth in vivo

**Figure 7:**
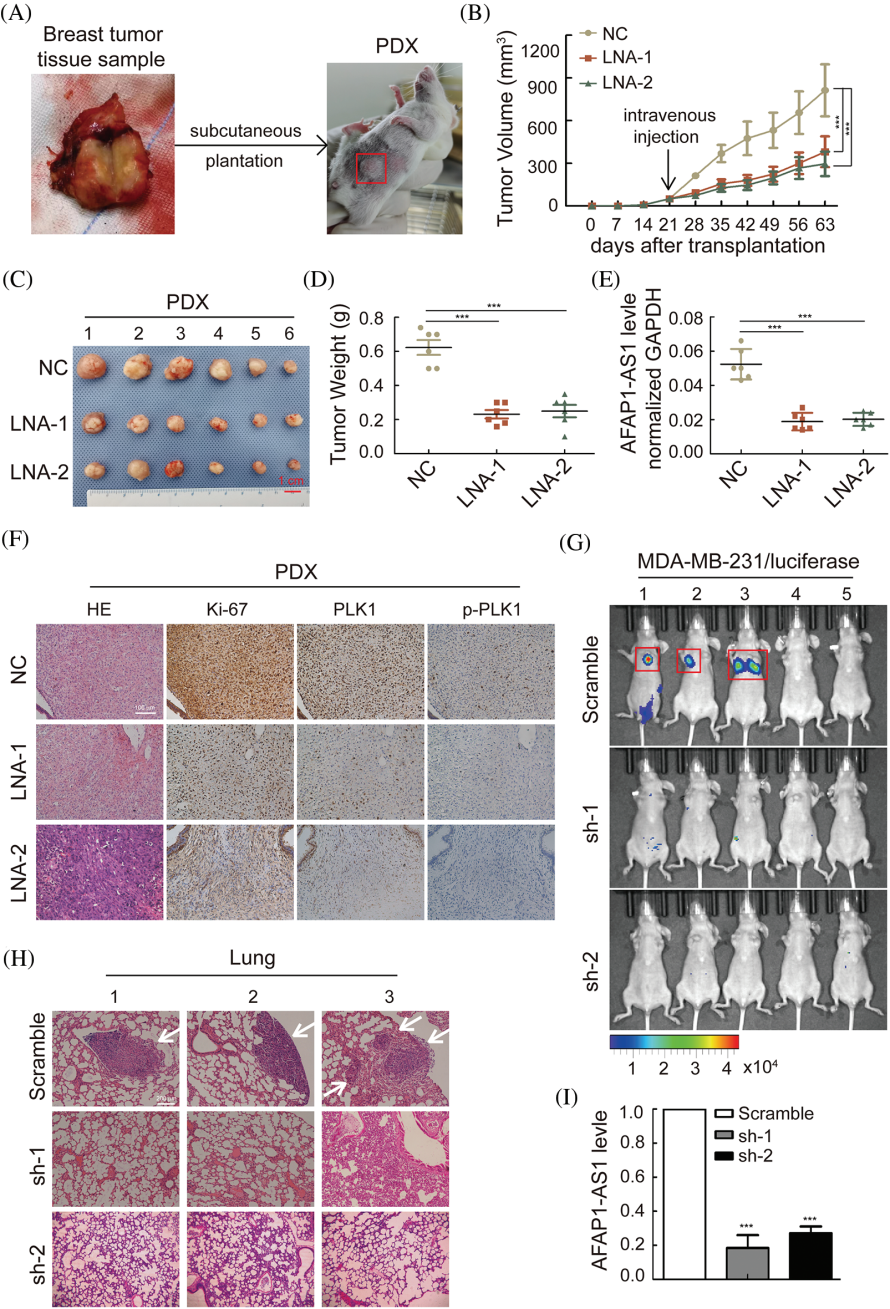
Knockdown of AFAP1-AS1 inhibited the proliferation and metastasis of TNBC *in vivo*. (A) The summarized procedure of the PDX model *in vivo* in this study. (B) Tumor size in different groups was calculated every 7 days over 2 months. (***p < 0.001 between the NC group and LNA groups by paired Student’s *t* tests, mean ± SD). (C) Representative images of PDXs in the different groups. AFAP1-AS1 knockdown by LNA inhibited tumor growth (scale bar: 1 cm). (D) The tumor weight of the representative images of 4C with AFAP1-AS1 knockdown by LNA was reduced compared to the NC group. (E) The expression of AFAP1-AS1 was decreased in the knockdown groups by LNA compared to the NC group. (F) PDX tissue sections were subjected to HE and IHC staining against Ki-67 and p-PLK1. Ki-67 and p-PLK1 were decreased in the knockdown groups by LNA compared to the NC group. (G) To explore the influence of AFAP1-AS1 on TNBC metastasis, live imaging *in vivo* was conducted by intravenous injection of MDA-MB-231 cells with luciferase, and the metastases in the lung were significantly fewer in the sh-AFAP1-AS1 groups than in the scramble group. (H) Representative lung metastasis nodes in the scramble group by HE staining (scale bar: 200 μm). (I) The efficiency of AFAP1-AS1 stable knockdown was reduced compared to that of the scramble group.

According to the above *in vitro* work, we established a TNBC PDX growth model in NOSCID mice to test whether *AFAP1-AS1* knockdown can prevent TNBC growth. A summary of the PDX establishment process is described in Fig. 7A. The tumor growth and weight of PDXs were significantly inhibited upon intravenous injection of *AFAP1-AS1* LNA compared to those of mice treated with control LNAs (Figs. 7B–7D). The efficiency of *AFAP1-AS1* knockdown by LNA *in vivo* was verified by qRT‒PCR (Fig. 7E). Ki-67, PLK1 and p-PLK1 staining by IHC showed that targeting *AFAP1-AS1* inhibited the proliferation and PLK1 phosphorylation of TNBC PDX *in vivo* (Fig. 7F).

### Knockdown of AFAP1-AS1 inhibited TNBC metastasis in vivo

To confirm whether *AFAP1-AS1* can affect TNBC metastasis, we injected MDA-MB-231 cells stably knocked out with sh-*AFAP1-AS1* lentivirus into the tail veins of BALB/c nude mice. After two months, the 6 mice in the scramble group experienced lung metastases in 3 mice, which did not occur in the sh-*AFAP1-AS1* groups, as determined by a live imaging system (Fig. 7G). HE staining in lung tissues of the scramble group showed significantly positive areas in metastasis node sections in contrast to the sh-*AFAP1-AS1* groups (Fig. 7H). The efficiency of *AFAP1-AS1* knockdown in different groups of lung tissues was proven by qRT‒PCR (Fig. 7I). Altogether, we concluded that *AFAP1-AS1* promoted TNBC growth and metastasis *in vivo*.

## Discussion

It has been generally accepted that the dysfunction of some lncRNAs plays a pivotal role in the malignant process of cancers, such as cancer proliferation, metastasis and mitosis [26]. It has been reported that *AFAP1-AS1* is involved in the malignant progression of a number of kinds of tumors and could be a prognostic biomarker in cancer. In this study, we explored the expression and functional role of *AFAP1-AS1* in TNBC primary cells and a PDX model. We found that *AFAP1-AS1* is highly expressed in TNBC tissues and primary cells and correlates with poor OS and DFS (MFS and RFS). We also found that silencing *AFAP1-AS1* expression in TNBC primary cells obviously induced apoptosis by facilitating mitotic catastrophe, and *AFAP1-AS1* promoted lung metastases *in vivo*. Thus, we further verified that *AFAP1-AS1* is a key oncogenic biomarker and that *AFAP1-AS1* is crucial for the survival of TNBC. *AFAP1-AS1* will possibly become an important therapeutic target for combating TNBC.

In our study, we found that *AFAP1-AS1* was upregulated in our TNBC cohort including 368 pairs of TNBC patients and correlated with outcome and disease progression. In our TNBC primary cell *in vitro* and *in vivo* models. We also found that *AFAP1-AS1* promoted TNBC migration, invasion, and proliferation and inhibited apoptosis. A previous study reported that *AFAP1-AS1* was upregulated and indicated poor prognosis in TNBC patients with radio resistance. *AFAP1-AS1* knockdown in cooperation with radiotherapy inhibited proliferation, migration and invasion and induced apoptosis in a TNBC cell line [19]. Interestingly, we discovered that *AFAP1-AS1* might participate in mitosis to inhibit TNBC primary cell death.

Here, we reported that *AFAP1-AS1* promoted TNBC growth by inhibiting mitotic catastrophe. In cancer, mitotic catastrophe is usually caused by genomic susceptibility, physical radiation and anticancer drugs [8]. The appearance of micronuclei and/or multinuclei is a characteristic early hint. Ultimately, mitotic catastrophe can be triggered by early-stage entry into mitosis [27]. Mitotic catastrophe can occur in a variety of diseases, especially in cancer [28]. The relationship between lncRNAs and mitotic catastrophe has not been reported. In our study, we found a higher proportion of micro- and multinuclei in TNBC primary cells, thereby inducing TNBC primary cell death when *AFAP1-AS1* was knocked out. *AFAP1-AS1* is known as an explicit oncogene in cancer, and our results will compensate for the lack that *AFAP1-AS1* can regulate TNBC mitosis.

We further explored the regulatory mechanism between *AFAP1-AS1* and mitosis. Some studies have shown that PLK1 is a member of the Polo-like kinase family and is a key mitotic kinase that localizes to kinetochores, centrosomes, and the central spindle and midbody [29]. PLK1 is mainly involved in the G1/M transition of cell cycle division by regulating centrosome maturation, disjunction and microtubule attachment [30]. Tumor cells will cause mitotic catastrophe and apoptosis in a variety of cancers when PLK1 activities are inhibited [31]. Some studies have shown that PLK1 is obviously overexpressed in TNBC compared with other BC subtypes [5]. In our study, by conducting gain- or loss-of-function assays, we demonstrated that the PLK1 pathway was inactivated by *AFAP1-AS1* knockdown but activated by *AFAP1-AS1* overexpression to influence the mitosis of TNBC primary cells rather than other mitosis-regulating genes, including HSET and Eg5 [32]. It is worth noting that lncRNA APAL regulates PLK1 activation and is involved in mitotic catastrophe in BC [31]. Their results also found one lncRNA involved in mitotic catastrophe influencing death in BC cells, similar to our results. In the future, additional studies are necessary to explore the precise regulatory mechanism of the *AFAP1-AS1* and PLK1 pathways in TNBC cells.

Accumulating data have demonstrated that *AFAP1-AS1* can regulate malignant biological behaviors such as migration, invasion, and proliferation in various tumors. In our study, we established a TNBC metastasis model *in vivo* and found that *AFAP1-AS1* knockdown by LNAs obviously inhibited lung metastasis of TNBC. In our clinical cohort, TNBC patients with high *AFAP1-AS1* expression had a higher metastasis rate than TNBC patients with low *AFAP1-AS1* expression. Bi et al. [19] also used a lung metastasis model *in vivo* and demonstrated that *AFAP1-AS1* silencing enhanced the radiotherapy effect to inhibit lung metastasis tumor growth. In Zhang et al.’s study, *AFAP1-AS1* promoted TNBC cell line growth, but data on *AFAP1-AS1* and metastases *in vivo* were not reported [18]. Overall, we found that *AFAP1-AS1* silencing decreased lung metastasis and inhibited PDX growth *in vivo*. Therefore, our results demonstrated that *AFAP1-AS1* could be an excellent prognostic gene and therapeutic target for TNBC.

In conclusion, *AFAP1-AS1*, as a key protumor biomarker, is upregulated in TNBC tissues and primary cells. The overexpression of *AFAP1-AS1* positively correlated with poor prognosis and high relapse and metastasis rates in TNBC patients. *AFAP1-AS1* promoted proliferation, migration and invasion, inhibited mitotic catastrophe in TNBC primary cells, and accelerated tumor growth in TNBC PDX and lung metastasis by activating PLK1. Our study represents a new molecular mechanism of *AFAP1-AS1* via PLK1 activity in TNBC carcinogenesis. The interaction of *AFAP1-AS1* and PLK1 is crucial in regulating malignant progression and the treatment of TNBC patients.

## Data Availability

The datasets used and/or analyzed during this study are available from the corresponding author on reasonable request.

## Abbreviations

TNBC: Triple-negative breast cancer
PLK1: Polo-like kinase 1
ISH: *In situ* hybridization
FISH: Fluorescence *in situ* hybridization
OS: Overall survival
DFS: Disease-free survival
MFS: Metastasis-free survival
RFS: Recurrence-free survival
IF: Immunofluorescence
PDX: Patient-derived xenograft
BC: Breast cancer
Her2: Human epidermal growth factor receptor 2
lncRNAs: Long noncoding RNAs
FA: Fibroadenoma
IHC: Immunohistochemical staining
NC: Negative control
qRT‒PCR: Real-time quantitative reverse transcription
WB: Western blotting
95% CI: 95% confidence interval
HR: Hazard ratio
CCK-8: Cell count Kit-8

## Appendix

**Table S1:** The clinical information of primary cells and PDX in this study

Patients ID	Age	Histology grade	Tumor size (cm)	Ki-67 (%)
758029 (FA-case1)	37	FA	1.0	–
757858 (FA-case2)	23	FA	1.6	–
758335 (FA-case3)	34	FA	2.9	–
757093 (TNBC-case1)	52	TNBC	3.3	80
741313 (TNBC-case2)	37	TNBC	2.9	85
755294 (TNBC-case3)	44	TNBC	3.1	85
744895 (TNBC-case4)	60	TNBC	3.3	75
735137 (TNBC-case5)	56	TNBC	3.9	70
742419 (PDX)	53	TNBC	4.1	90

**Table S2:** Univariate and Multivariate Cox proportional hazard analyses of OS and DFS in 368 TNBC patients

Variable	OS				DFS			
	Univariate analysis		Multivariate analysis		Univariate analysis		Multivariate analysis	
	HR (95% CI)	*p*	HR (95% CI)	*p*	HR (95% CI)	*p*	HR (95% CI)	*p*
AFAP1	2.149 (1.522–3.035)	0.000	2.149 (1.522–3.035)	0.0	3.687 (2.583–5.262)	0.000	3.687 (2.583–5.262)	0.0
Age	0.987 (0.973–1.001)	0.062	–	–	0.997 (0.984–1.010)	0.683	–	–
Menopausal status	0.603 (0.429–0.847)	0.004	Not selected	Not selected	0.987 (0.718–1.359)	0.938	–	–
Tumor size	0.974 (0.889–1.067)	0.574	–	–	0.954 (0.863–1.054)	0.354	–	–
Histological grade	1.058 (0.825–1.357)	0.656	–	–	0.998 (0.794–1.254)	0.986	–	–
Ki-67 level	1.004 (0.998–1.010)	0.144	–	–	1.004 (0.998–1.009)	0.188	–	–
